# The Tryptophan Operator Acts as a Portable Downstream Enhancer for Recombinant Protein Expression in *Escherichia coli*

**DOI:** 10.3390/ijms27219589

**Published:** 2026-10-27

**Authors:** Buli Su, Peixuan Lai, Xiong Xiong, Yonghong Wang, Man Li, Honghui Zhu, Ming-Rong Deng

**Affiliations:** Guangdong Microbial Culture Collection Center, Guangdong Provincial Observation and Research Station for Microbial Science of the Ecosystems of Origin for Geographical Indication Agricultural Products, Key Laboratory of Agricultural Microbiomics and Precision Application (MARA), Key Laboratory of Agricultural Microbiome (MARA), State Key Laboratory of Applied Microbiology Southern China, Institute of Microbiology, Guangdong Academy of Sciences, Guangzhou 510070, China

**Keywords:** *trpO*, tryptophan operator, enhancer, recombinant protein expression, *Escherichia coli*, L-threonine

## Abstract

Downstream enhancer-like regulatory elements remain largely unexplored in prokaryotes. The tryptophan operator sequence (*trpO*), an 18-bp near-palindromic element from the *Escherichia coli trpEDCBA* operon, is conventionally defined as the binding site for the TrpR repressor, which blocks transcription initiation. Here, we report a previously unrecognized function of *trpO*: it can serve as a portable, orientation-dependent downstream enhancer that increases recombinant protein expression in *E. coli*. Inserting *trpO* downstream of multiple constitutive promoters increased eGFP reporter expression, with the reverse orientation (*retrpO*) conferring the most prominent enhancement, yielding a 1.56-fold expression increase. Adding L-threonine further raised expression to 3.98-fold. After screening plasmid replicons and host strains, the combination of the CloDF13 replicon and W3110 host, together with L-threonine supplementation, achieved a cumulative 9.57-fold enhancement in protein expression. Together, these findings challenge the long-standing notion that trpO functions exclusively as a negative regulatory element and establish trpO as a portable tool for increasing protein yields in *E. coli*.

## 1. Introduction

*Escherichia coli* is widely used for recombinant protein production because of its rapid growth, well-characterized genetics, and ease of genetic manipulation. Heterologous expression is regulated by multiple transcriptional and translational regulatory elements, including promoters, ribosome-binding sites (RBSs), untranslated regions (UTRs), and transcription terminators. Promoter engineering has dominated efforts to optimize expression, generating extensive libraries of constitutive and inducible promoters with graded transcriptional strengths [1]. Recent studies have identified the 5′UTR as an equally important determinant of expression, because its secondary structure, nucleotide composition, and spacer length can each alter bacterial translation efficiency by orders of magnitude [2,3,4]. The conserved start element near the transcription start site (TSS) influences both TSS selection and transcriptional activity across diverse bacterial phyla, and the spacer between the −35 and −10 elements alone can tune transcription up to 600-fold [5].

In contrast to the extensive engineering of upstream promoters and 5′UTRs, enhancer-like sequences located downstream of the TSS that stimulate expression remain largely unexplored in bacteria. Eukaryotic enhancers are well characterized, whereas their prokaryotic counterparts are rarer and operate through different mechanisms. Classic bacterial UP elements enhance transcription by interacting directly with RNA polymerase in core-promoter upstream regions [6], whereas σ^54^-dependent systems rely on protein-mediated DNA looping from distant sites [7]. To our knowledge, no short, portable DNA sequence that increases expression from a downstream position has been characterized in bacteria. Two recent findings nevertheless point to regulatory potential in this region. Gene-less cassettes in *Vibrionaceae* superintegrons carry internal promoter activity [8], and deep-learning-designed full-length promoters can predictably tune transcription [9].

The *E. coli* tryptophan (*trp*) operon is a classic bacterial regulatory system governed by two well-characterized layers of control. Repression is mediated by the TrpR repressor, which binds the *trpO* operator upon association with tryptophan and blocks transcription initiation [10]. Attenuation is mediated by the *trpL* leader peptide, which couples translation to transcription termination [11]. For half a century, the 18-bp *trpO* sequence has been understood exclusively as a negative regulatory element, and no activating or enhancing function has been attributed to it.

TrpR is a homodimeric helix–turn–helix protein that is inactive as an apoprotein and becomes competent to bind DNA only when two molecules of L-tryptophan bind, non-cooperatively, at the dimer interface [12,13]. Its co-repressor is therefore L-tryptophan, and only ligands that combine an indole ring with an amino group activate the repressor [14]; no other amino acid has been reported to bind TrpR. TrpR controls three operons in *E. coli* [15], and its operator is built from two half-sites in an inverted repeat [16]; all characterized functions of these sites are repressive. Earlier work indicates that amino acids other than tryptophan can influence *trp* expression, although it measured derepression of the *trp* biosynthetic enzymes in minimal medium rather than the behavior of the operator itself. L-threonine alone raised the specific activity of anthranilate synthetase about 1.4-fold, whereas phenylalanine with tyrosine gave 3.4-fold and a mixture of six amino acids about fivefold, so the response is not specific to L-threonine [17].

During our investigation of L-threonine-mediated regulation of the *trp* operon, we unexpectedly observed that insertion of *trpO* downstream of the synthetic constitutive promoter P*J23119* increased reporter gene expression even in the absence of L-threonine. This finding, in which a repressor-binding site exerted an activating rather than a repressive effect, contrasts with the established role of *trpO* and prompted us to characterize its regulatory properties systematically. Here, we show that *trpO* acts as an orientation-dependent downstream element that increases expression from each of the three constitutive promoters, three plasmid backbones, and three *E. coli* host strains tested. We further show that L-threonine supplementation amplifies *trpO*-mediated enhancement, and that combined optimization of the *retrpO* configuration and expression chassis produces a 9.57-fold cumulative improvement in protein production over the initial baseline.

## 2. Results

### 2.1. trpO Enhances Recombinant Protein Expression Across Multiple Promoters

**Figure 1:**
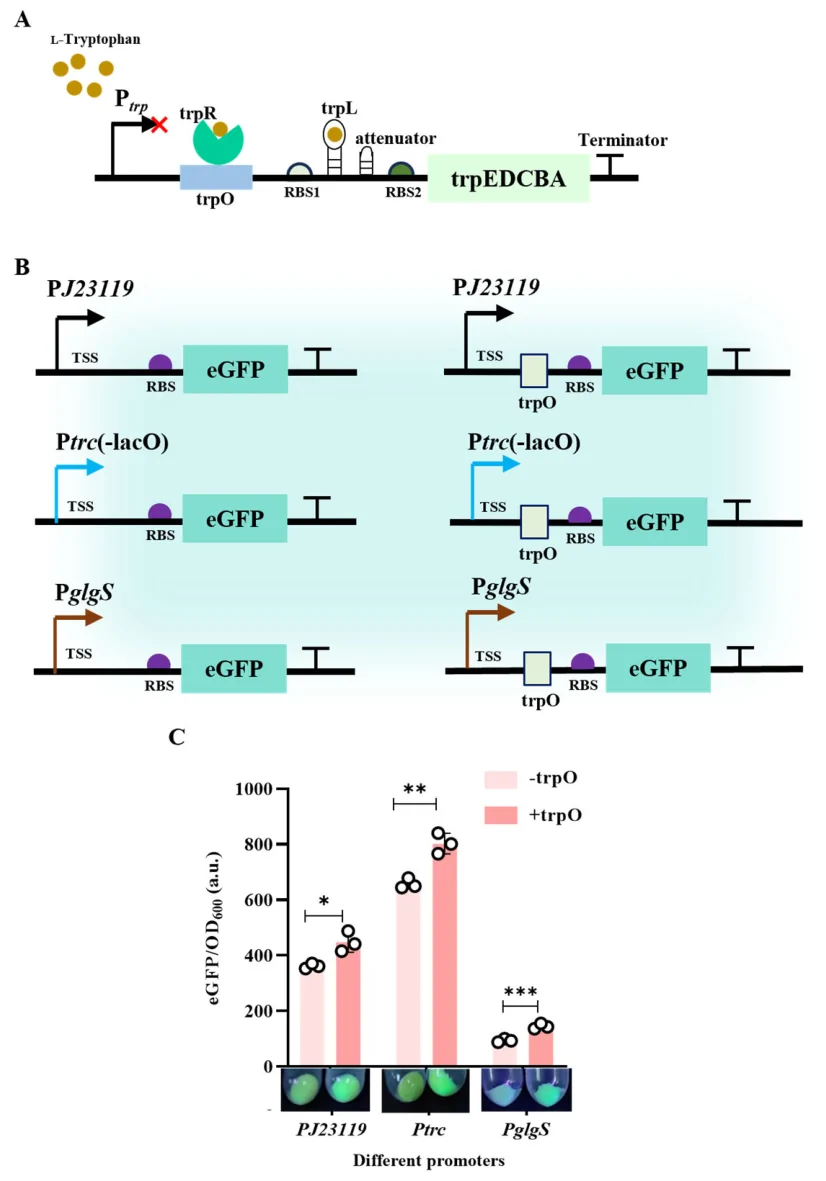
Regulation of the native *trp* operon and enhancement of eGFP expression by *trpO* inserted downstream of the transcription start site (TSS) across different constitutive promoters. (**A**) Regulation of the native *trp* operon. The trpO operator lies upstream of *trpE* and is bound by the TrpR-L-tryptophan holorepressor, which blocks transcription initiation, while attenuation at the *trpL* leader controls transcription read-through into *trpE*. (**B**) Schematic of the expression modules with different promoters. (**C**) Relative fluorescence normalized to OD_600_ for three promoters (P*J23119*, P*trc(-lacO)*, and P*glgS*) with and without *trpO* inserted in the forward orientation downstream of the TSS. Data are means ± SD (n = 3 biological replicates). * *p* < 0.05, ** *p* < 0.01 and *** *p* < 0.001 by two-tailed Student’s *t*-test.

To determine whether *trpO*-mediated enhancement depends on promoter sequence, we inserted *trpO* in the forward orientation downstream of three constitutive promoters (P*J23119*, P*trc(-lacO)*, and P*glgS*) spanning a range of intrinsic strengths. The three promoters differ in origin. P*J23119* is a synthetic promoter, P*trc(-lacO)* a *lacO*-deleted variant of the *trc* promoter, and P*glgS* the promoter of the *E. coli glgS* gene. The same *egfp* reporter and the same pACYC backbone were used for all constructs. The 18-bp element was placed at an identical position in each, between the transcription start site and the ribosome-binding site. Each *trpO* construct was measured alongside a promoter-only control carrying the same promoter and reporter, so that the only difference between the two was the presence of the insert. All *trpO*-containing constructs produced significantly higher eGFP fluorescence than the corresponding promoter-only controls (Figure 1).

### 2.2. Reverse Orientation of trpO Confers the Greatest Enhancement

**Figure 2:**
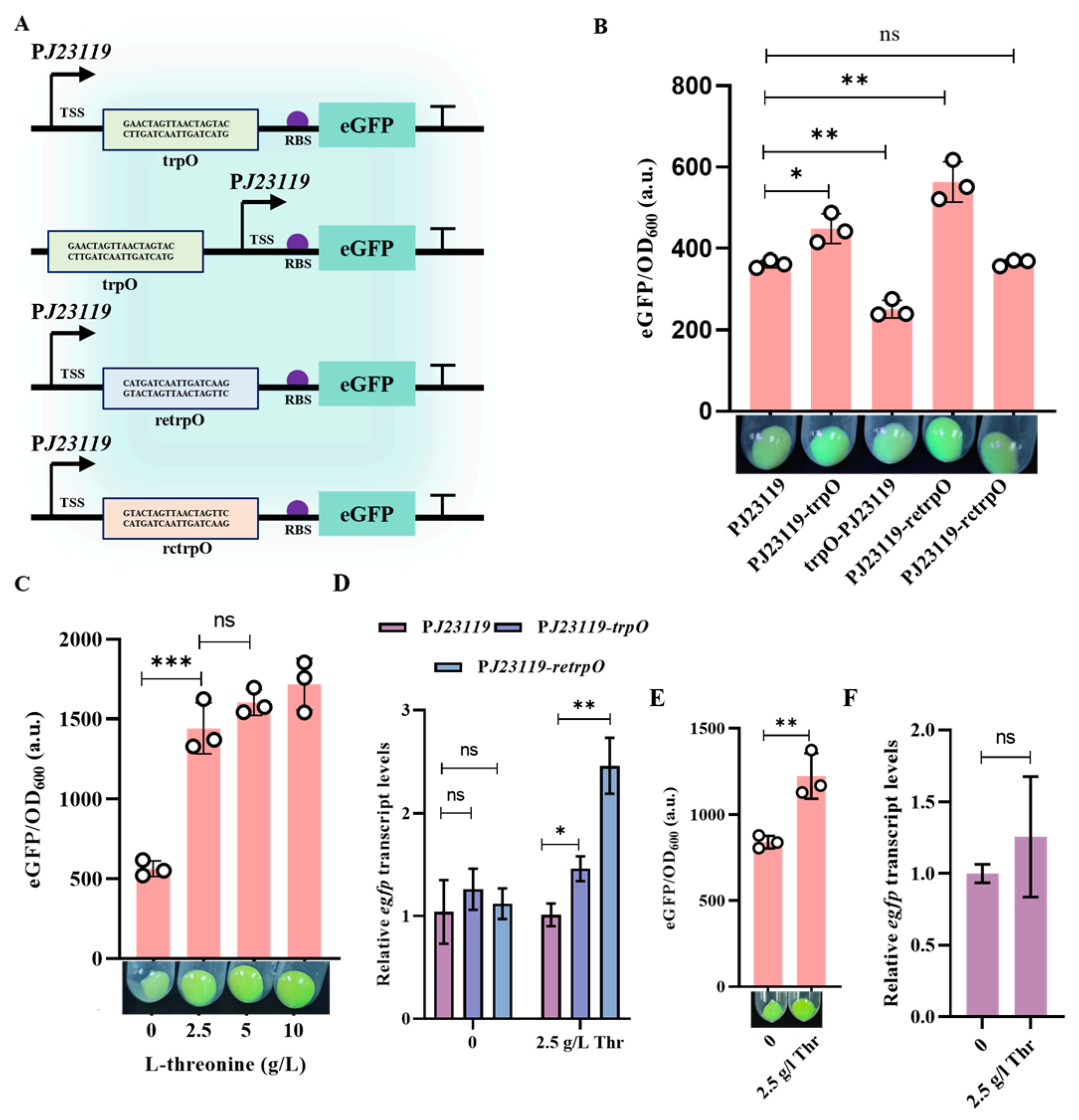
Effect of *trpO* configuration on eGFP expression from the P*J23119* promoter. (**A**) Schematic of the four tested configurations: *trpO* upstream of the promoter (*trpO*-P*J23119*), forward downstream (P*J23119*-*trpO*), reverse downstream (P*J23119*-*retrpO*), and reverse-complement downstream (P*J23119*-*rctrpO*). (**B**) Normalized fluorescence of the four configurations without L-threonine. (**C**) Normalized fluorescence of the PJ23119-retrpO construct at different L-threonine concentrations. (**D**) Relative *egfp* transcript levels of the P*J23119*, P*J23119*-*trpO*, and P*J23119*-*retrpO* configurations with and without L-threonine, determined by RT-qPCR. (**E**) Normalized fluorescence of the P*J23119-retrpO* construct in the *ΔtrpR* derivative of W3110 without and with 2.5 g/L L-threonine. (**F**) Relative *egfp* transcript levels of the same strain under the same two conditions, determined by RT-qPCR. Data are means ± SD (n = 3 biological replicates). * *p* < 0.05, ** *p* < 0.01 and *** *p* < 0.001, and ns (not significant) by two-tailed Student’s *t*-test.

To define the positional and orientational requirements for *trpO*-mediated expression enhancement, we systematically compared four configurations around the P*J23119* promoter: upstream (*trpO*-P*J23119*), forward downstream (P*J23119*-*trpO*), reverse downstream (P*J23119*-*retrpO*), and reverse-complement downstream (P*J23119*-*rctrpO*). All four inserts are 18 bp long, and in the three downstream constructs they occupy the same position between the TSS and the RBS, so the TSS-to-RBS distance is identical among them and 18 bp longer than in the promoter-only control. The interval between the TSS and the RBS sets the sequence and folding context of the 5′UTR and can by itself change expression by more than two orders of magnitude [18,19]. Comparing the three downstream constructs, which have identical spacing, therefore isolates the effect of the inserted sequence from the effect of the spacing change. Only the two downstream placements containing the native *trpO* sequence enhanced eGFP expression relative to the promoter-only control. The reverse orientation produced the largest increase (1.56-fold; Figure 2A,B). Upstream insertion reduced expression, indicating that the effect requires the downstream position as well as the *trpO* sequence. The reverse-complement orientation had no significant effect, even though *rctrpO* has the same length and the same base composition as *trpO* (6 G + C and 12 A + T residues out of 18) and differs from it at only two of the eighteen positions (2 and 17). The three downstream constructs therefore differed only in the sequence of the inserted 18-bp element, and the enhancement tracked that sequence rather than any generic property of an AT-rich insert. These controls exclude insert length, base composition, and spacing as the cause of the enhancement.

Supplementation with 2.5 g/L L-threonine further increased *retrpO*-driven eGFP expression to 3.98-fold relative to the promoter-only control, with no additional benefit at higher concentrations (Figure 2C). RT-qPCR analysis showed that this further increase was accompanied by higher *egfp* transcript abundance. In the absence of L-threonine, *egfp* transcript levels were similar among the P*J23119*, P*J23119*-*trpO*, and P*J23119*-*retrpO* constructs. After addition of 2.5 g/L L-threonine, transcript levels increased in both *trpO*-containing constructs, with the *retrpO* group showing the strongest upregulation (Figure 2D).

To ask whether this L-threonine-dependent increase in transcript abundance requires *TrpR*, we introduced the same construct into a Δ*trpR* derivative of W3110 and measured eGFP fluorescence and *egfp* transcript abundance alongside the parental strain (Figure 2E,F). Basal fluorescence was higher in Δ*trpR* than in W3110. Supplementation with 2.5 g/L L-threonine still increased eGFP expression in Δ*trpR*, but by a much smaller margin than in W3110. Transcript levels behaved differently: in Δ*trpR*, they did not differ significantly with and without L-threonine, whereas the parental strain responded as shown in Figure 2D. The L-threonine-dependent increase in transcript abundance therefore requires *TrpR*.

### 2.3. CloDF13 Replicon and W3110 Host Yield the Highest trpO-Mediated Expression

**Figure 3:**
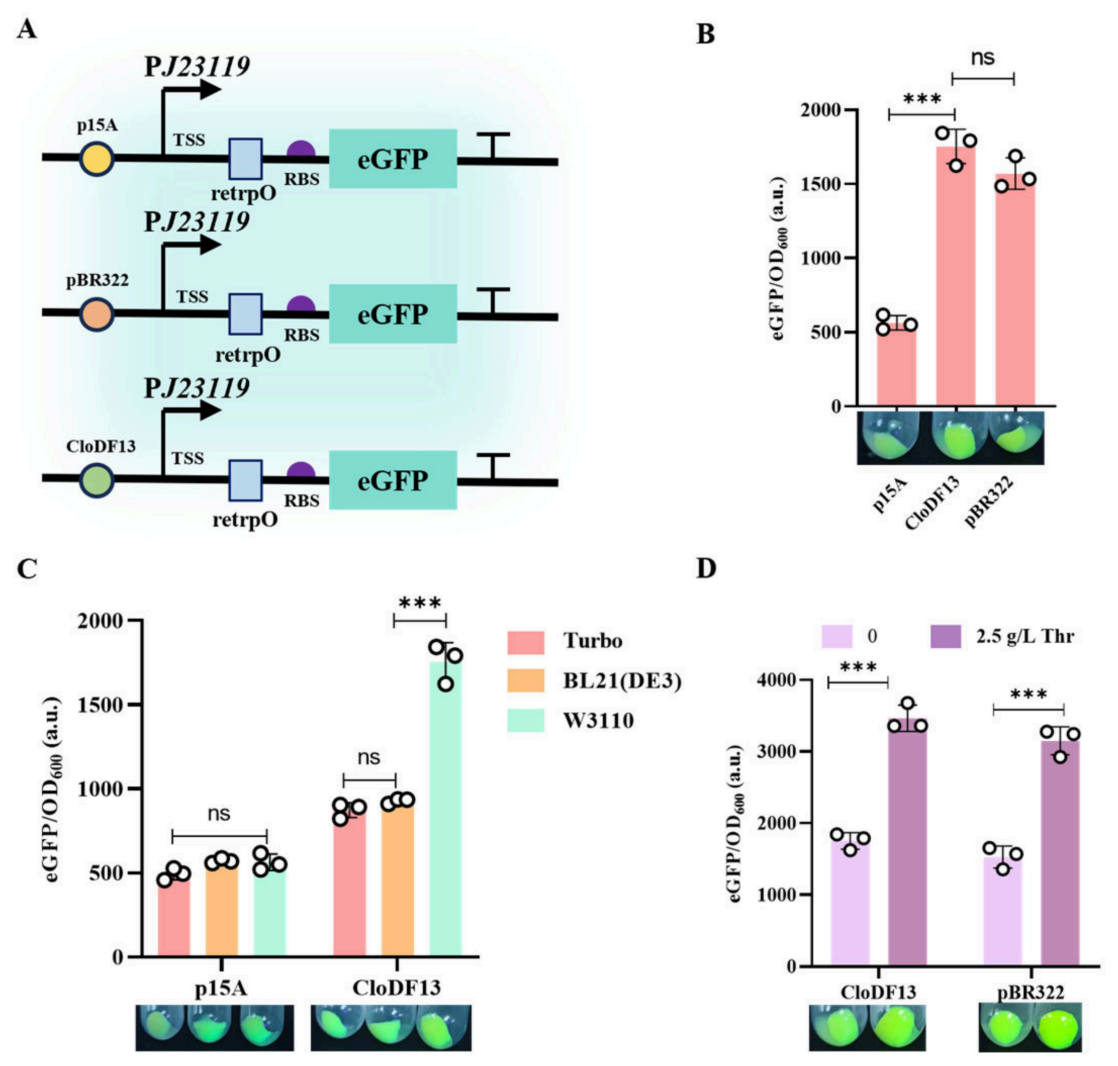
Optimization of the expression chassis for P*J23119*-*retrpO*-eGFP. (**A**) Expression modules with different replicons. (**B**) Effect of plasmid replicons (p15A, CloDF13, and pBR322) on eGFP expression in *E. coli* W3110. (**C**) Effect of host strains (Turbo, BL21(DE3), and W3110) on eGFP expression using the p15A and CloDF13 replicons. (**D**) Normalized fluorescence with L-threonine supplementation using the CloDF13 and pBR322 replicons in *E. coli* W3110. Data are means ± SD (n = 3 biological replicates). *** *p* < 0.001 and ns (not significant) by two-tailed Student’s *t*-test.

To identify an optimal expression chassis, we tested the best-performing construct (P*J23119*-*retrpO*-eGFP) across three plasmid replicons and three *E. coli* host strains. Among the replicons, CloDF13 gave the highest fluorescence (Figure 3A,B). Among the host strains, the K-12 derivative W3110 produced the highest expression (Figure 3C). In this experiment, the same P*J23119*-*retrpO*-eGFP construct was compared across hosts, so the values in Figure 3C represent absolute expression levels. The CloDF13/W3110 combination with L-threonine supplementation produced a 6.15-fold improvement over the initial P*J23119*-*retrpO* configuration (Figure 3D). The fully optimized system, consisting of P*J23119*-*retrpO*-eGFP on the CloDF13 replicon in W3110 with L-threonine supplementation, achieved a cumulative 9.57-fold enhancement over the initial promoter-only baseline without modifying the core promoter or introducing exogenous regulatory proteins. This value combines changes made in separate experiments: within a single standardized genetic background the *trpO* element contributes 1.56-fold, L-threonine supplementation raises this to 3.98-fold, and the optimized replicon and host strain account for the remainder.

## 3. Discussion

Together, these results show that *trpO* raises the expression of a downstream gene without any modification of the promoter. The increase is a property of the *trpO* sequence rather than of inserting 18 bp at that position. At the same site the native element enhanced expression, whereas its reverse complement, which differs at only two of eighteen positions (2 and 17) and leaves the spacing unchanged, had no detectable effect. The enhancement has two components that differ in their dependence on L-threonine.

The L-threonine-independent component acts after transcription. In the absence of L-threonine, *trpO*-containing constructs reached higher fluorescence than their promoter-only controls without a measurable change in transcript abundance. This places the component at the level of translation or mRNA stability rather than at transcription initiation. Because *trpO* lies between the transcription start site and the ribosome-binding site, it is transcribed into the 5′UTR of the *egfp* mRNA, where the AT-rich element can fold into a stem-loop. Such structures protect mRNA from exonucleolytic degradation and alter translation efficiency. 5′UTR structure can change mRNA half-life from approximately 20 s to more than 20 min [2]. Stem-loops have likewise been linked to increased mRNA stability [4,20].

The L-threonine-dependent component is superimposed on the first when the amino acid is supplied, and behaves differently. It is accompanied by an increase in *egfp* transcript abundance and therefore includes a change at the level of transcription. It is unlikely to have a direct ligand effect on TrpR, because the co-repressor site accepts only indole-bearing analogues of tryptophan [14]. L-threonine is not one, so this component must act through the *trp* regulatory system.

The *trp* regulatory system does, however, respond to L-threonine. In our earlier work on an L-threonine biosensor, reported separately, L-threonine was shown to derepress the tryptophan-repressed *trp* promoter [21]. There, the *trpO* operator and the *trpL* leader sequence both contributed to the response, and combining the two with the otherwise constitutive P*J23119* promoter gave the lowest background and the clearest L-threonine-dependent induction, matching the native promoter. Deleting *trpR* in that study raised basal expression and left a response only at high L-threonine concentrations. In the present constructs the deletion reproduced the first half of that pattern: basal expression rose, and the L-threonine-dependent increase in transcript abundance disappeared, leaving only a smaller response that does not involve transcription. We propose that TrpR, rather than an unidentified threonine-responsive factor, is therefore the protein that mediates the L-threonine response at low concentrations. Of the twenty proteinogenic amino acids, only L-threonine elicited a detectable response, and L-tryptophan suppressed reporter expression, consistent with its role as the cognate co-repressor. A general response to the amino-acid content of the medium, such as relief of the stringent response that amino-acid limitation triggers [22], would not be confined to one amino acid. The L-threonine dependence seen here is therefore the same regulatory behavior observed previously in a different construct, and *trpO* is the element that confers it.

The molecular step that links L-threonine to TrpR has not been identified. The most economical explanation of the link is a fall in the intracellular L-tryptophan pool, so that less holorepressor is formed and *trpO* is less occupied. Deaminated tryptophan analogues occupy the co-repressor site in a flipped orientation and derepress the *trp* operon in the same way [15], showing that tryptophan-responsive regulation can be perturbed without L-threonine binding TrpR at all. In the *trp* operon, *trpO* represses transcription, whereas here it enhances expression of a gene placed downstream of the transcription start site. The positive direction of the effect is a consequence of that position, which places the element in the transcript rather than over the promoter. Repressor sites are known to occupy that position in *E. coli*, but the characterized examples, such as the *PurR* site in *purB*, are repressive [23]. The present data establish two features: the enhancement requires the *trpO* sequence at the downstream position and not merely an 18-bp insert; one of the two components is controlled by L-threonine, and that component requires *TrpR*.

The consistent effect across promoters of different strengths indicates that the enhancement does not require a particular promoter sequence, and distinguishes *trpO* from architecture-dependent bacterial activators, including the UP-elements and σ^54^-dependent enhancers [7,24]. Recent efforts have focused on engineering 5′UTR elements to enhance expression independently of promoter sequence. For example, modification of the 5′ region of a fusion-tag mRNA can balance transcript abundance and ribosome-binding energy, improving soluble recombinant-protein titers by 1.3- to 5.3-fold [20]. Rational design of 5′UTRs with defined spacer lengths and GC content allows predictable tuning of expression [4], whereas eliminating cytidine from the 5′UTR substantially increases bacterial translation efficiency [3]. In contrast to these strategies, which require optimization of nucleotide composition, length, or folding energy, *trpO* is a compact 18-bp element whose sequence requires no additional optimization*. trpO* is also considerably smaller than the gene-less cassettes identified in *Vibrionaceae* superintegrons, which contain internal promoters [8]. Together with the conserved start element [5] and de novo-designed insulated cis-regulatory elements [9], these findings identify the post-TSS region as an underused target for bacterial gene-expression engineering, with *trpO* providing a modular element for this purpose.

Elements that increase expression from a downstream position have also been described outside bacteria. The yeast TCIN5B system repurposes transcription-factor binding sites, placed downstream of the TEF1 promoter, to increase expression by up to 15.25-fold [25]. Native CsrA-binding sequences in the *glgC* 5′UTR have likewise been rewired into synthetic post-transcriptional regulatory modules [26]. Because the magnitude of enhancement depends on the promoter, the reporter, the host, and the culture format, these values place *trpO* in a similar range to the systems above. The relevant features of *trpO* are that it is a single 18-bp element, insertable downstream of an existing promoter without altering the promoter itself. Its contribution can also be increased by supplementing the medium with L-threonine. L-threonine is produced industrially by fermentation and is therefore available on a bulk scale. Supplementing it at 2.5 g/L nevertheless adds a raw-material cost that must be weighed against the additional gain.

The superiority of CloDF13 is consistent with a favorable balance between gene dosage and metabolic burden at its moderate-to-high copy number. Host suitability is product-dependent. BL21 outperforms W3110 for soluble scFv production [27], but W3110 was clearly superior for the eGFP reporter used here. The cumulative 9.57-fold enhancement is the product of fold-changes measured in separate experiments. The reverse orientation contributed 1.56-fold, L-threonine supplementation raised this to 3.98-fold, and chassis optimization brought the total to 9.57-fold. *trpO*-mediated enhancement can also be combined with other expression-optimization strategies, such as ribosomal protein S1 engineering [28], AI-guided promoter design [9], or cross-species expression platforms [29].

## 4. Materials and Methods

### 4.1. Strains, Plasmids, and Culture Conditions

*Escherichia coli* strains Turbo, BL21(DE3), and W3110 were used as heterologous expression hosts. BL21(DE3) is deficient in the Lon and OmpT proteases [27] and carries the λDE3 lysogen expressing T7 RNA polymerase under the lacUV5 promoter [30], whereas W3110 is a K-12 derivative [31]. All strains were cultured in LBMS medium (5 g/L tryptone, 2.5 g/L yeast extract, 5 g/L NaCl, and 5 g/L MOPS) at 37 °C with shaking at 200 rpm. Where indicated, L-threonine was added to final concentrations of 2.5, 5, or 10 g/L. Antibiotic selection used chloramphenicol (34 μg/mL) for pACYC-derived plasmids, kanamycin (50 μg/mL) for pET30a-derived plasmids, and spectinomycin (50 μg/mL) for pCDF-derived plasmids. The three vector backbones compared in this work differ in their replication origins and therefore in copy number: pACYC carries the p15A origin [32], pET30a the pBR322 origin [33], and pCDF the CloDF13 origin [34]. The Δ*trpR* derivative of W3110 was described previously [21]. The complete list of strains, plasmids, and primers is provided in Tables S1 and S2.

### 4.2. Construction of Promoter-trpO-egfp Reporter Plasmids

The enhanced green fluorescent protein (eGFP) gene, a FACS-optimized GFP variant [35], was used as the reporter throughout. Three constitutive promoters were tested: P*J23119*, a synthetic promoter from the Anderson promoter collection [36]*,* P*trc*(-*lacO*), a *lacO*-deleted derivative of the *trc* promoter [37], and P*glgS*, the promoter of the *E. coli glgS* gene [38]. Each promoter was cloned upstream of *egfp* in the pACYC vector backbone [32] using the ClonExpress MultiS One Step Cloning Kit (Vazyme, Nanjing, China) according to the manufacturer’s protocol. The promoter sequence, the position of the insert and the architecture of every construct are given in Table 1, and the complete sequences of these parts are listed in Table S3. Four configurations of the *trpO* sequence (5′-GAACTAGTTAACTAGTAC-3′) were constructed relative to the P*J23119* promoter and its transcription start site (TSS): (i) upstream of the promoter, 5′ of the −35 element (*trpO-*P*J23119*); (ii) in the forward orientation between the TSS and RBS (P*J23119-trpO*); (iii) in the reverse orientation between the TSS and RBS (P*J23119-retrpO*; sequence, 5′-CATGATCAATTGATCAAG-3′); and (iv) in the reverse-complement orientation at the same position (P*J23119-rctrpO*; sequence, 5′-GTACTAGTTAACTAGTTC-3′). In every construct, the 18-bp element was added between the promoter and the ribosome-binding-site segment rather than substituted for any part of them. For each promoter, the promoter-only control and the corresponding element-containing construct were amplified from the same template with primer pairs that share an identical promoter-side arm and an identical RBS-side arm, the element being introduced as an extension on the inner end of the forward primer. The two amplicons therefore contain the same promoter sequence and the same RBS segment, with 18 bp inserted between them. Consequently, relative to its own promoter-only control, each element-containing construct has an unchanged promoter sequence, an unchanged TSS position and an unchanged RBS segment, and a TSS-to-RBS distance that is exactly 18 bp longer (Table 1). All variants were generated by whole-plasmid PCR with the primers listed in Table S2. Each PCR product was digested with DpnI (Vazyme, Nanjing, China) to remove the methylated template, phosphorylated with T4 polynucleotide kinase (Takara Bio, Kusatsu, Japan), and self-ligated with T4 DNA ligase (Takara Bio, Kusatsu, Japan). Ligation mixtures were individually transformed into chemically competent *E. coli* Turbo cells. Positive clones were selected on LB agar containing the appropriate antibiotic and verified by Sanger sequencing.

### 4.3. Fluorescence Measurement

Single colonies were inoculated into 5 mL of LB medium containing antibiotics and incubated at 37 °C and 200 rpm for 12 h to prepare overnight seed cultures. Each seed culture was diluted 1:100 into 30 mL of fresh LBMS medium in a 150 mL baffled shake flask, supplemented with L-threonine at the indicated concentration where applicable, and grown at 37 °C and 200 rpm for 24 h. At harvest, 200 μL of culture was transferred to a black-walled, clear-bottom 96-well microplate. Fluorescence (excitation, 488 nm; emission, 509 nm; bandwidth, 5 nm) and OD_600_ were recorded with a Thermo Scientific Varioskan LUX multimode microplate reader (Thermo Fisher Scientific, Waltham, MA, USA). Relative fluorescence was expressed as arbitrary fluorescence units normalized to OD_600_ (eGFP/OD_600_).

### 4.4. RT-qPCR Analysis

At the 4 h time point, 1 mL of each culture was harvested by centrifugation (12,000× *g*, 2 min, 4 °C). Total RNA was extracted with the HiPure Bacterial RNA Kit (Magen, Guangzhou, China), including an on-column DNase I digestion to remove residual genomic DNA. First-strand cDNA was synthesized from 500 ng of total RNA with the HiScript III RT SuperMix for qPCR (Vazyme, Nanjing, China) and random hexamer primers. Quantitative PCR was performed on a QuantStudio 6 PCR Detection System (Applied Biosystems, Waltham, MA, USA) with ChamQ Universal SYBR qPCR Master Mix (Vazyme, Nanjing, China). *egfp* transcripts were detected with primers qPCR-eGFP-F and qPCR-eGFP-R. The 16S rRNA gene served as the internal reference and was amplified with qPCR-16S-F and qPCR-16S-R. Thermal cycling conditions were: 95 °C for 30 s; 40 cycles of 95 °C for 5 s and 60 °C for 30 s; followed by a melt curve analysis. Relative transcript levels were calculated by the 2^−ΔΔCt^ method.

### 4.5. Plasmid Replicon Screening and Host Strain Screening

To test the effect of copy number on *trpO*-mediated enhancement, the P*J23119*-*retrpO*-*egfp* expression cassette was moved from the pACYC backbone, which carries the p15A replicon, into two alternative backbones: a pET30a derivative carrying the pBR322 replicon and a pCDF derivative carrying the CloDF13 replicon. Assembly, transformation, and sequence verification followed the procedures described above. The P*J23119*-*retrpO-egfp* construct in each replicon backbone was introduced into chemically competent *E. coli* Turbo, BL21(DE3), and W3110 cells, prepared in-house by the standard CaCl_2_ method. Transformation, antibiotic selection, and fluorescence measurement were performed as described above.

## 5. Conclusions

For the first time, we demonstrate that *trpO*, long recognized as the binding site of the TrpR repressor, also increases expression of a gene placed downstream of it. The effect depends on both the sequence and the orientation of the inserted element. The reverse orientation outperformed the native forward configuration, whereas a reverse-complement sequence inserted at the same position, which has the same length and the same base composition as *trpO* and differs from it at only two of eighteen positions, had no detectable effect. The activity therefore cannot be attributed to insert length, to base composition, or to the increased TSS-to-RBS distance. Combining the best-performing *trpO* configuration, chassis engineering, and L-threonine supplementation produced a cumulative 9.57-fold enhancement over the initial baseline under the conditions tested. The L-threonine-dependent component of this enhancement requires TrpR; when *trpR* is deleted, the increase in *egfp* transcript abundance disappears. These findings indicate that a classical bacterial operator sequence can serve as a compact cis-acting element for recombinant protein production.

## Figures and Tables

**Table 1:** Architecture of the reporter constructs. Every construct carries a promoter, an optional 18-bp insert, the same ribosome-binding-site segment (5′-TAACTTTAAGAAGGAGATATACAT-3′), the *egfp* coding sequence and a terminator in the pACYC backbone. In each element-containing construct the 18-bp insert was added between the promoter and the ribosome-binding-site segment, so that relative to its promoter-only control the promoter sequence, the transcription start site and the ribosome-binding-site sequence are unchanged and the distance between the transcription start site and the ribosome-binding site is exactly 18 bp longer. Within the P*J23119* series, the three downstream constructs carry the insert at the same position and therefore have identical spacing.

Construct	Promoter	Insert (5′ → 3′)	Insert Position
P*J23119*-eGFP (control)	P*J23119*	none	—
P*J23119*-*trpO*	P*J23119*	GAACTAGTTAACTAGTAC	TSS–RBS
P*J23119*-*retrpO*	P*J23119*	CATGATCAATTGATCAAG	TSS–RBS
P*J23119*-*rctrpO*	P*J23119*	GTACTAGTTAACTAGTTC	TSS–RBS
*trpO*-P*J23119*	P*J23119*	GAACTAGTTAACTAGTAC	upstream of the promoter
P*trc*(-*lacO*)-eGFP (control)	P*trc*(-*lacO*)	none	—
P*trc*(-*lacO*)-*trpO*	P*trc*(-*lacO*)	GAACTAGTTAACTAGTAC	TSS–RBS
P*glgS*-eGFP (control)	P*glgS*	none	—
P*glgS*-*trpO*	P*glgS*	GAACTAGTTAACTAGTAC	TSS–RBS

## Data Availability

The original contributions presented in this study are included in the article/Supplementary Materials. Further inquiries can be directed to the corresponding authors.

## Supplementary Materials

The following supporting information can be downloaded at https://www.mdpi.com/article/10.3390/ijms27219589/s1.
