# Supplementary material for: The Tryptophan Operator Acts as a Portable Downstream Enhancer for Recombinant Protein Expression in Escherichia coli
Source: Int J Mol Sci. 2026 Oct 27;27(21):9589. doi: 10.3390/ijms27219589 (PMC13762905; doi:10.3390/ijms27219589)
Supplement: Supplementary file 1 [file ijms-27-09589-s001.zip › ijms-4645725-supplementary.pdf]

Supporting Information

The tryptophan operator acts as a portable downstream enhancer for recombinant protein expression in *Escherichia coli*

Buli Su, Peixuan Lai, Xiong Xiong, Yonghong Wang, Man Li, Honghui Zhu*, Ming-Rong Deng*

Guangdong Microbial Culture Collection Center, Guangdong Provincial Observation and Research Station for Microbial Science of the Ecosystems of Origin for Geographical Indication Agricultural Products, Key Laboratory of Agricultural Microbiomics and Precision Application (MARA), Key Laboratory of Agricultural Microbiome (MARA), State Key Laboratory of Applied Microbiology Southern China, Institute of Microbiology, Guangdong Academy of Sciences, Guangzhou 510070, People's Republic of China

*Corresponding author

Ming-Rong Deng: E-mail: dengmr@gdim.cn

Honghui Zhu: E-mail: zhuhh_gdim@163.com

Tel: +86-020-87137635, Fax: +86-020-87685635

Table S1 Strains and plasmids used in this study.

Strain/plasmid	Description	Source
Strains		
Turbo	*F' proAB lacIq lacZΔM15 / fhuA2 (lac-proAB) glnV galK16 galE15 R(zgb-210::Tn10) TetS endA1 thi-1 Δ(hsdS-mcrB)*	Lab stock
W3110	Wild type, *λ- F- mcrA mcrB IN (rrnD-rrnE)1*	Lab stock
BL21(DE3)	*F-ompT hsdSB (rB-mB-) gal dcm (DE3)*	Lab stock
ΔtrpR	W3110, ΔtrpR	[21]
Plasmids		
pACYCDuet-1	P15A-derived vector; Cm^r	Lab stock
pCDFDuet-1	CloDF13-derived vector; Sm^r	Lab stock
pET30a	pBR322-derived vector; Km^r	Lab stock
pACYC-trpL-eGFP	pACYCDuet-1 plasmid with *eGFP* under P_{trpL} promoter	Lab stock
pACYC-PJ23119-eGFP	pACYCDuet-1 plasmid with eGFP under P_{J23119} promoter	Lab stock
pACYC-PJ23119-trpO-eGFP	pACYCDuet-1 plasmid with *eGFP* under P_{J23119} promoter, and with trpO	This study
pACYC-trc(-lacO)-eGFP	pACYCDuet-1 plasmid with *eGFP* under P_{trc} promoter without lacO	This study
pACYC-trc(-lacO)-trpO-eGFP	pACYCDuet-1 plasmid with *eGFP* under P_{trc} promoter without lacO, and with trpO	This study
pACYC-PglgS-eGFP	pACYCDuet-1 plasmid with *eGFP* under P_{glgS} promoter	This study
pACYC-PglgS-trpO-eGFP	pACYCDuet-1 plasmid with *eGFP* under P_{glgS} promoter, and with trpO	This study
pACYC-trpO-PJ23119-eGFP	pACYCDuet-1 plasmid with *eGFP* under P_{J23119} promoter, and with trpO upstream of the promoter	This study
pACYC-PJ23119-retrpO-eGFP	pACYCDuet-1 plasmid with *eGFP* under P_{J23119} promoter, and with the reverse trpO	This study
pACYC-PJ23119-rctrpO-eGFP	pACYCDuet-1 plasmid with *eGFP* under P_{J23119} promoter, and with the reverse complement trpO	This study
pCDF-PJ23119-retrpO-eGFP	pCDFDuet-1 plasmid with *eGFP* under P_{J23119} promoter, and with the reverse trpO	This study
pET30a-PJ23119-retrpO-eGFP	pET30a plasmid with *eGFP* under P_{J23119} promoter, and with the reverse trpO	This study

Sm^r, streptomycin; Cm^r, chloramphenicol; Km^r, kanamycin; R, resistance.

Table S2. Primers used in this study.

Primers	Sequence (5' to 3')
**Construction of pACYC-PJ23119-trpO-eGFP**	
pJ23119-trpL-F0305	ttgacagctagctcagtcctaggtataatgctagcacgtaaaaagggtatcgacaatgaaagc
pJ23119-ACYC-R0114	aggactgagctagctgtcaaacttcaggtgctacatttgaag
pJ23119-trpL-F0330	taggtataatgctagcgaactagttaactagtacacgtaaaaagggtatcgaca
pJ23119-R0330	gtgtactagttaactagttcgctagcattatacctta
PtrpO-eGFP-F	taactttaagaaggagatatacatatggtga
pJ23119-trpO-(-trpL) eGFP-R0402	gtatatctccttcttaaagttagtactagttaactagttcgct
**Construction of pACYC-trc(-lacO)/-trpO-eGFP**	
trc(-lacO)-eGFP-F0418	caaatgtagcacctgaagtgttgacaattaatcatccggctcgtataatgtgtggaataactttaagaaggagatatacat
trc-(-lacO)-eGFP-R0418	gccccaaggggttatgctagctacttgtacagctcgtccatg
pACYC-F0418	ctagcataaccccttggggcct
pACYC-R0418	acttcaggtgctacatttgaa
trc-trpO-eGFP-F0418	ctcgtataatgtgtggaagaactagttaactagtactaactttaagaaggagatatac
trc-trpO-eGFP-R0418	ttccacacattatacgagccggatgat
**Construction of pACYC-PglgS/-trpO-eGFP**	
PglgS-F0519	taaggggtgttatagtcggcga
PglgS-R0519	gcataccttttaaggccatatg
pACYC-trpO-eGFP-F0519	tatggccttaaaaggtatgcgaactagttaactagtactaactttaa
pACYC-eGFP-R0519	cgccgactataacaccccttaacttcaggtgctacatttgaag
**Different trpO**	
trpO-PJ23119-F0422	gaactagttaactagtacttgacagctagctcagtcctagg
trpO-PJ23119-R0422	tgtcaagtactagttaactagttcacttcaggtgctacatttgaag
PJ23119-retrpO-F0422	cctaggtataatgctagccatgatcaattgatcaagtaactttaagaaggagatataca
PJ23119-retrpO-R0422	gctagcattatacctaggactgagctagctgtcaa
PJ23119-rctrpO-F0422	cctaggtataatgctagcgtactagttaactagttctaactttaagaaggagatataca
**Different plasmid ori**	
PJ23119-eGFP-F0429	ttgacagctagctcagtcctagg
trpL-eGFP-R0401	caaaaaacccctcaagacccg
pET30a-trpL-F0401	ggtcttgaggggttttttgctgaaaggaggaactatatccg
pET30a-PJ23119-R0429	taggactgagctagctgtcaatgactatcgtcgccgcacttatg
pCDF-PJ23119-R0429	taggactgagctagctgtcaagcggttcagtagaaaagatcaaag
**qPCR**	
qPCR-eGFP-F	catgaagcagcacgacttcttc
qPCR-eGFP-R	cttgaagttcaccttgatgccg
qPCR-16S-F	ggggagcaaacaggattagata
qPCR-16S-R	atgtcaagaccaggtaaggttc

Table S3. The DNA sequences of different biological parts.

Biological parts	Sequence (5' to 3')
P*J23119*	ttgacagctagctcagtcctaggtataatgctagc
P*trc(-lacO)*	gttgacaattaatcatccggctcgtataatgtgtggaa
P*glgS*	taaggggtgttatagtcggcgaataaaatcatatatcactccaatgtccatttagtgacgcaata<br>tttactttgtaatagtttccttgcttacatttatctgtgttaataataacacaggttagagaagttctg<br>gaagagaaagtcttttaatatggtttttatttttcactaaaaagtgtgatcggggacaatatattta<br>cgcacgttatgtttaaaggcactacactgattggggaatactgaaatcagaaaaagcatatgg<br>ccttaaaaggtatgc
RBS	taactttaagaaggagatatacat
eGFP	atggtgagcaagggcgaggagctgttcaccggggtggtgcccatcctggtcgagctggac<br>ggcgacgtaaacggccacaagttcagcgtgtccggcgagggcgagggcgatgccaccta<br>cggcaagctgaccctgaagttcatctgcaccaccggcaagctgcccgtgccctggcccacc<br>ctcgtgaccaccctgacctacggcgtgcagtgcttcagccgctaccccgaccacatgaagc<br>agcacgacttcttcaagtccgccatgcccgaaggctacgtccaggagcgcaccatcttcttca<br>aggacgacggcaactacaagacccgcgccgaggtgaagttcgagggcgacaccctggtg<br>aaccgcatcgagctgaagggcatcgacttcaaggaggacggcaacatcctggggcacaag<br>ctggagtacaactacaacagccacaacgtctatatcatggccgacaagcagaagaacggca<br>tcaaggtgaacttcaagatccgccacaacatcgaggacggcagcgtgcagctcgccgacc<br>actaccagcagaacacccccatcggcgacggccccgtgctgctgcccgacaaccactacct<br>gagcacccagtccgccctgagcaaagaccccaacgagaagcgcgatcacatggtcctgct<br>ggagttcgtgaccgccgccgggatcactctcggcatggacgagctgtacaagtag
T7 terminator	ctagcataaccccttggggcctctaaacgggtcttgaggggttttttg
p15A ori	tttccataggctccgcccccctgacaagcatcacgaaatctgacgctcaaatcagtggtggcg<br>aaacccgacaggactataaagataccaggcgtttccccctggcggctccctcgtgcgctctcct<br>gttcctgcctttcggtttaccggtgtcattccgctgttatggccgcgtttgtctcattccacgcctg<br>acactcagttccgggtaggcagttcgctccaagctggactgtatgcacgaaccccccgttca<br>gtccgaccgctgcgccttatccggtaactatcgtcttgagtccaacccggaaagacatgcaaa<br>agcaccactggcagcagccactggtaattgatttagaggagttagtcttgaagtcatgcgccg<br>gttaaggctaaactgaaaggacaagttttggtgactgcgctcctccaagccagttacctcggtt<br>caaagagttggtagctcagagaaccttcgaaaaaccgccctgcaaggcggttttttcgttttca<br>gagcaagagattacgcgcagaccaaaacgatctcaa
pBR322 ori	ttgagatcctttttttctgcgcgtaatctgctgcttgcaaacaaaaaaaccaccgctaccagcgg<br>tggtttgtttgccggatcaagagctaccaactctttttccgaaggtaactggcttcagcagagc<br>gcagataccaaatactgtccttctagtgtagccgtagttaggccaccacttcaagaactctgta<br>gcaccgcctacatacctcgctctgctaatcctgttaccagtggctgctgccagtggcgataagt<br>cgtgtcttaccgggttggactcaagacgatagttaccggataaggcgcagcggtcgggctga<br>acggggggttcgtgcacacagcccagcttggagcgaacgacctacaccgaactgagatac<br>ctacagcgtgagctatgagaaagcgccacgcttcccgaagggagaaaggcggacaggtat<br>ccggtaagcggcagggtcggaacaggagagcgcacgagggagcttccagggggaaacg<br>cctggtatctttatagtcctgtcgggtttcgccacctctgacttgagcgtcgatttttgtgatgctc<br>gtcaggggggcggagcctatggaaa
CloDF13 ori	ttccgtggataagcaggggactaacatgtgaggcaaaacagcagggccgcgccggtggcg

	tttttccataggctccgcccctcctgccagagttcacataaacagacgcttttccggtgcatctgt<br>gggagccgtgaggctcaaccatgaatctgacagtacgggcgaaacccgacaggacttaaa<br>gatccccaccgtttccggcgggtcgctccctcttgcgctctcctgttccgaccctgccgtttac<br>cggatacctgttccgcctttctcccttacgggaagtgtggcgctttctcatagctcacacactgg<br>tatctcggctcggtgtaggtcgttcgctccaagctgggctgtaagcaagaactccccgttcag<br>cccgactgctgcgccttatccggtaactgttcacttgagtccaacccggaaaagcacggtaaa<br>acgccactggcagcagccattggtaactgggagttcgcagaggatttgtttagctaaacacg<br>cggttgctcttgaagtgtgcgccaaagtccggctacactggaaggacagatttggttgctgtg<br>ctctgcgaaagccagttaccacggttaagcagttccccaactgacttaaccttcgatcaaacc<br>acctccccaggtggtttttcgtttacagggcaaaagattacgcgcagaaaaaaaggatctca<br>agaagatcctttga
